# MRI tracking of autologous pancreatic progenitor-derived insulin-producing cells in monkeys

**DOI:** 10.1038/s41598-017-02775-0

**Published:** 2017-05-31

**Authors:** Chunlin Zou, Yi Lu, Xiahong Teng, Shuyan Wang, Xiaoting Sun, Fen Huang, Guannan Shu, Xin Huang, Hongwei Guo, Zhiguo Chen, Jian Zhang, Yu Alex Zhang

**Affiliations:** 1 Center for Translational Medicine, Guangxi Medical University, Guangxi, P.R. China; 2 Cell Therapy Center, Xuanwu Hospital, Capital Medical University, Beijing, P.R. China; 3 Key Laboratory of Longevity and Ageing-related Diseases, Ministry of Education, Guangxi Medical University, Guangxi, P.R. China; 4 Guangxi Nanning Wincon TheraCells Biotechnologies Co., Guangxi, P.R. China

## Abstract

Insulin-producing cells (IPCs) derived from a patient’s own stem cells offer great potential for autologous transplantation in diabetic patients. However, the limited survival of engrafted cells remains a bottleneck in the application of this strategy. The present study aimed to investigate whether nanoparticle-based magnetic resonance (MR) tracking can be used to detect the loss of grafted stem cell-derived IPCs in a sensitive and timely manner in a diabetic monkey model. Pancreatic progenitor cells (PPCs) were isolated from diabetic monkeys and labeled with superparamagnetic iron oxide nanoparticles (SPIONs). The SPION-labeled cells presented as hypointense signals on MR imaging (MRI). The labeling procedure did not affect the viability or IPC differentiation of PPCs. Importantly, the total area of the hypointense signal caused by SPION-labeled IPCs on liver MRI decreased before the decline in C-peptide levels after autotransplantation. Histological analysis revealed no detectable immune response to the grafts and many surviving insulin- and Prussian blue-positive cell clusters on liver sections at one year post-transplantation. Collectively, this study demonstrates that SPIO nanoparticles can be used to label stem cells for noninvasive, sensitive, longitudinal monitoring of stem cell-derived IPCs in large animal models using a conventional MR imager.

## Introduction

Type 1 diabetes (T1D) is a disorder of carbohydrate metabolism characterized by hyperglycemia. T1D is caused by cell-mediated autoimmune destruction of pancreatic β-cells. T1D can lead to a variety of severe complications, including retinopathy, nephropathy, and cardiovascular diseases. Although exogenous insulin injection is effective in lowering blood glucose levels in T1D patients, it does not restore the physiological regulation of blood glucose. Consequently, insulin-based therapy delays but cannot prevent the onset and progression of diabetic complications^1, 2^. In addition, intensive insulin therapy increases the risk of potentially life-threatening hypoglycemia^3^. Pancreatic islet transplantation has recently been validated as a promising approach to restore physiological secretion of insulin in T1D patients^4–6^. However, the broad application of islet replacement therapy is significantly hampered by a limited source of cadaveric islets, and many studies have suggested that stem cell-derived insulin-producing cells could serve as an alternative source of functional islet cells^7–9^. In particular, recent advances in cell reprogramming and large-scale generation of functional β-cells from human pluripotent stem cells have enabled an unlimited source of β-ells for autologous transplantation in T1D^10^.

One of the major limitations of current β-cell transplantation approaches is the poor survival of grafts regardless of alloislet (http://www.citregistry.org/) or autoislet transplantation^11, 12^. Therefore, timely detection of the transplanted β-cells is important to maintain good glycemic control. Fasting blood glucose and C-peptide levels are often used as clinical parameters for assessing islet graft function but are not suitable for the sensitive and timely detection of islet graft loss because these two biochemical indices do not change significantly until decompensation of islet β-cells occurs. An ideal monitoring modality should permit early detection of islet damage to enable timely therapeutic intervention and protect the islet grafts from further damage. Superparamagnetic iron oxide nanoparticles are the most commonly used magnetic resonance imaging (MRI) contrast agent for biomedical imaging^13, 14^, and transplanted SPION-labeled cells can be visualized as hypointense areas by MRI^15–17^. Several previous preclinical and clinical studies have tested the feasibility and safety of SPION-based MR tracking of transplanted islets^12, 18–20^. However, these studies have not revealed the temporal relationship between islet graft dysfunction and loss of graft islet mass. A recent primate study by Wang *et al*. demonstrated that SPION-based MRI can detect graft volume reduction prior to any detectable changes in blood glucose after intrahepatic islet allotransplantation^21^. However, whether SPION-based MRI monitoring can be applied for the timely detection of graft loss following autologous stem cell-derived IPC transplantation remains unclear.

In the current study, we investigated the feasibility and efficacy of an *in vivo* MR tracking approach for longitudinally monitoring autologous stem cell-derived IPC grafts using a clinical 1.5-T MRI scanner in a cynomolgus diabetic model. Our data demonstrate that the SPION labeling procedure did not affect the viability or differentiation capacity of adult pancreatic progenitor cells *in vitro* and that our MRI tracking method can reliably detect IPC graft loss prior to graft dysfunction.

## Results

### Establishment of a stable T1D monkey model by streptozotocin treatment

**Figure 1:**
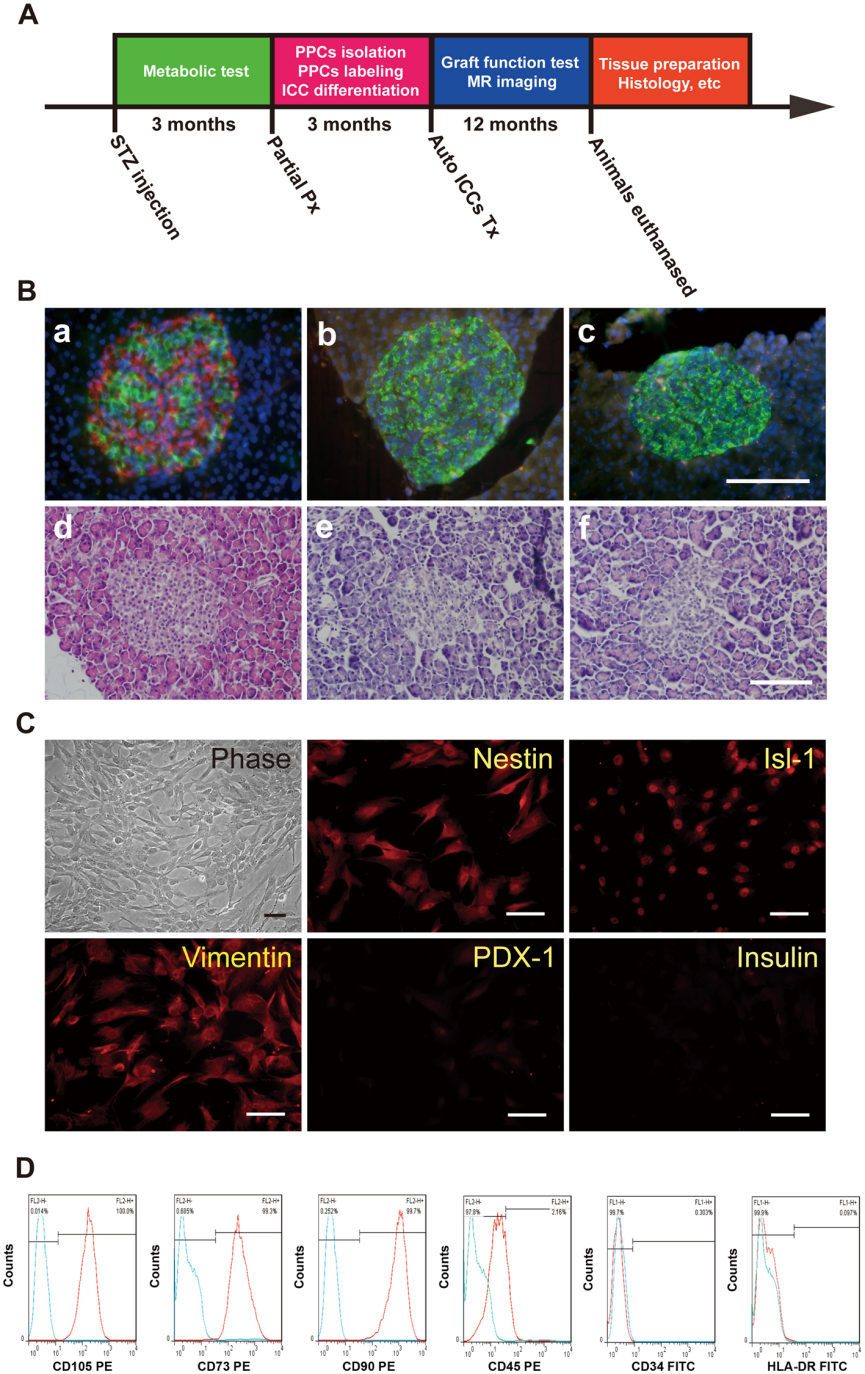
(**A**) A schematic diagram illustrating the experimental paradigm. (**B**) Immunostaining and H&E staining of pancreatic biopsies from normal monkeys (a, d), diabetic monkeys 3 months after STZ injection (b, e) and diabetic monkeys up to one year after implantation (c, f). In diabetic monkeys, insulin expression (red fluorescence) decreased, whereas glucagon expression (green fluorescence) appeared to have expanded. H&E staining showed that exocrine pancreatic cells in diabetic monkeys appeared histologically normal. (**C**) After 8 passages, the PSCs maintained classic MSC-like morphology, and immunofluorescence labeling showed that the PPCs were positive for nestin, Isl-1 and vimentin but negative for PDX-1 and insulin. (**D**) The PPCs exhibited a cell surface marker expression pattern similar to that of bone marrow-derived MSCs. Flow cytometry analysis showed that the PPCs were positive for CD105, CD73, and CD90 but negative for CD45, CD34, and HLA-DR. Scale bars represent 100 μm.

To evaluate the effect of autotransplantation with SPION-labeled ICCs in diabetic monkeys, we first established a stable T1D model by administration of 100 mg/kg streptozotocin (STZ). All monkeys that received STZ exhibited hyperglycemia within 2–3 days. In all nine diabetic monkeys, fasting blood glucose levels increased to >15 mM, and non-fasting blood glucose levels increased to >17 mM. These diabetic monkeys began to receive daily injections of insulin on day 2 post-STZ treatment to maintain stable metabolism and survival. To eliminate the possible effect of islet regeneration on blood glucose and insulin levels, fresh pancreatic tissues were obtained from the T1D animals by biopsy 3 months after STZ injection and after euthanasia. Double immunostaining of pancreatic islets for insulin and glucagon revealed very few insulin-positive cells in the residual islets of the pancreas in the diabetic monkeys, and the remaining islets consisted mainly of glucagon-expressing cells. By contrast, islets from normal monkeys showed a normal pattern of insulin and glucagon staining. Hematoxylin and eosin (H&E) staining demonstrated that exocrine pancreatic cells appeared histologically normal in diabetic monkeys (Fig. 1B).

### Characterization of pancreatic progenitor cells (PPCs)

We have previously reported that PPCs can be isolated from fresh pancreata of diabetic monkeys and expanded for more than 10 passages *in vitro* ^22^. Here, we examined the expression of pancreatic and stem cell markers in these progenitor cells by immunofluorescence staining. A majority of the PPCs expressed the neuroepithelial precursor cell marker nestin, the insulin gene enhancer protein ISL-1, and vimentin but scarcely expressed pancreas/duodenum homeobox protein 1 (PDX-1) and insulin (Fig. 1C). Furthermore, flow cytometric analyses showed that that the cell surface marker expression pattern of the PPCs was similar to that observed in bone marrow-derived MSCs. Specifically, the PPCs exhibited no expression of hematopoietic markers (CD34 and CD45) or the marker for lymphocytes and monocytes (HLA-DR). Nearly 100% of the PPCs expressed the typical MSC marker proteins CD105, CD73, and CD90 (Fig. 1D).

### Labeling of pancreatic progenitor cells with SPIONs

**Figure 2:**
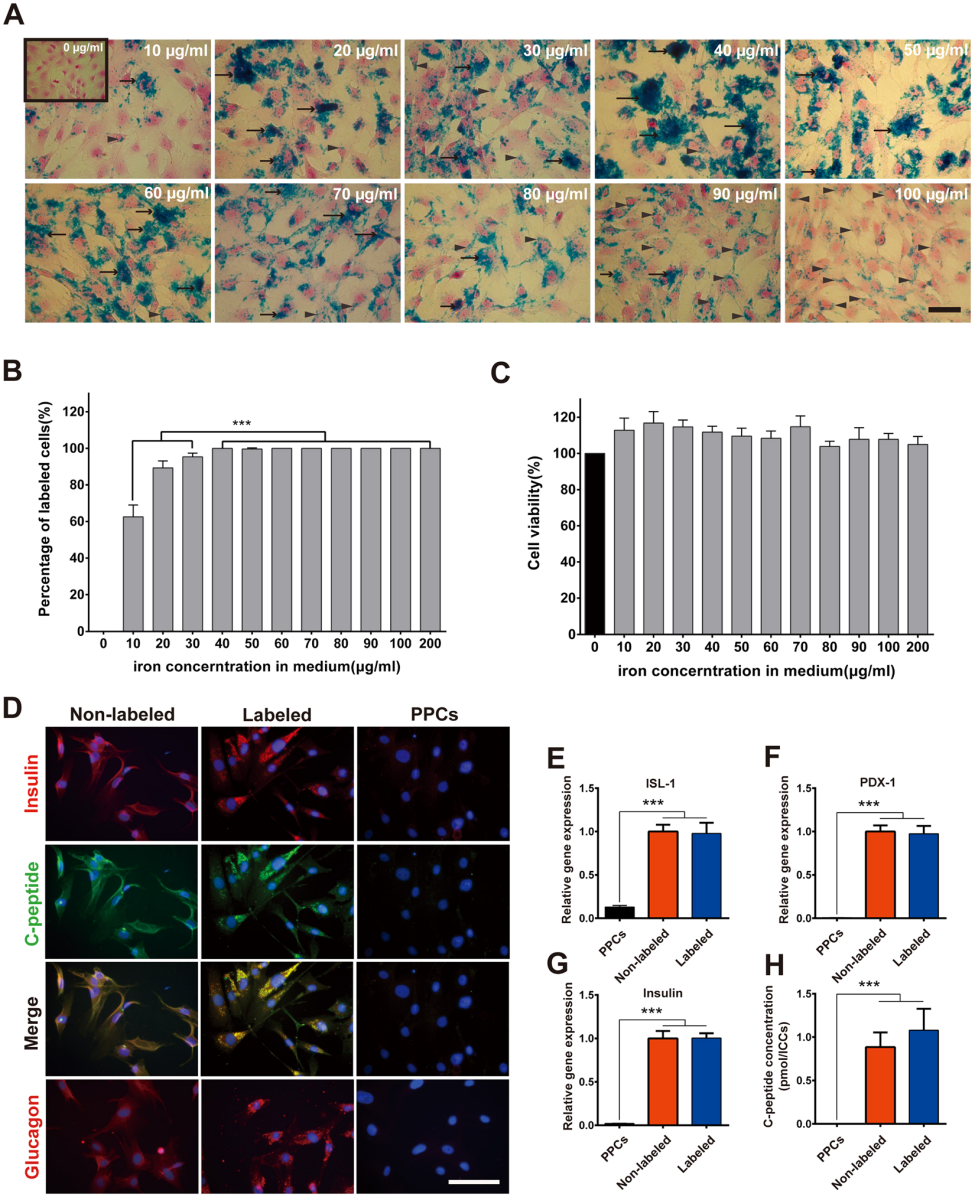
(**A**) Light micrographs showing the effect of SPION concentration on the uptake of iron oxide nanoparticles in cultured PPCs by Prussian blue staining. Few Prussian-blue positive cells were detected in the 10 μg Fe/ml SPIONs group, and more positive cells were present in the groups treated with concentrations of SPIONs of more than 20 μg Fe/ml. However, numerous Feridex-PLL complexes deposited on the cellular surface were observed in groups treated with 20 to 70 μg Fe/ml (arrows indicate Feridex-PLL complexes on the cellular surface). The deposition of complexes on the cellular surface was significantly decreased in groups treated with more than 80 μg Fe/ml SPIONs, and most of the Feridex-PLL complexes were phagocytized into the cytoplasm (arrowheads indicate Feridex-PLL complexes inside cells). These results suggested that 100 μg Fe/ml of SPIONs was an optimal concentration. (**B** and **C**) Bar graphs showing the effect of SPION concentration on the labeling efficiency and cell viability. The results are shown as the mean ± SD, n = 8 (independent experiments); ***P < 0.001. (**D**) In the final stage of differentiation, non-labeled and labeled cells showed expression of insulin (red fluorescence), C-peptide (green fluorescence), glucagon (red fluorescence), and colocalized insulin/C-peptide (orange fluorescence). PPCs were used as a negative control. Real-time PCR analysis of β-cell-specific genes: (**E**) Isl-1, (F) PDX-1, and (**G**) insulin expression in PPCs and non-labeled, and labeled ICCs. (**H**) ELISA measurements of the C-peptide content in PPCs and non-labeled, and labeled ICCs. The results shown are the mean ± SD, n = 4 (independent experiments); ***P < 0.001. Scale bars represent 100 μm.

To optimize the concentration range for labeling PPCs with SPIONs, we measured the labeling efficiency and cell viability across a range of SPION concentrations (0–200 μg Fe/ml). The optimal SPION concentration for *in vitro* labeling of PPCs was 100 μg Fe/ml. Although the labeling efficiency with SPIONs at a concentration of more than 40 μg Fe/ml was approximately 100%, many Feridex-poly-L-lysine (Feridex-PLL) complexes attached to the cellular membrane were observed at 20 to 70 μg Fe/ml. The deposition of the complexes onto the cellular membrane was significantly decreased when the concentration was greater than 80 μg Fe/ml, and at this concentration, most of the Feridex-PLL complexes were localized in the cytoplasm (Fig. 2A and B). Electron microscopy images confirmed that the Feridex-PLL complexes were mainly located in the cytoplasm of PPCs treated with 100 μg Fe/ml SPIONs. The unique appearance of SPIONs was markedly different from the morphology of other organelles, such as the nucleus, mitochondria and lysosomes (Supplementary Figure S1B). As shown in Figure S1C, the SPIONs were phagocytozed into the cytoplasm of PPCs. Next, we investigated whether SPIO labeling affected cell viability and β-cell differentiation capacity. The effect of SPIONs on the cell viability of PPCs was examined using an MTS assay. As illustrated in Fig. 2C, SPIONs in the concentration range of 10–200 Fe/ml had no effect on the cell viability of PPCs compared with the control (untreated) group.

### Effect of SPION labeling on the differentiation of PPC-derived ICCs

To confirm that SPION labeling did not alter the capability of PPCs to differentiate into pancreatic β-cells, *in vitro* studies were performed. After the PPCs were terminally differentiated into islet/β-like cell clusters (ICCs), the ICCs were fixed and labeled with antibodies specific to insulin and C-peptide. As shown in Fig. 2D, SPION labeling did not alter the expressions of the representative pancreatic endocrine markers, such as insulin and glucagon, in labeled or non-labeled cells. Moreover, all of the insulin-positive cells coexpressed C-peptide, indicating an undeniable production of insulin. Furthermore, the mRNA expression levels of Isl-1, Pdx-1 and insulin in PPCs, non-labeled ICCs and labeled ICCs were examined by real-time PCR. Compared with PPCs, non-labeled ICCs expressed 48-fold higher insulin mRNA levels, and Isl-1 and PDX-1 levels increased approximately 8-fold and 159-fold, respectively. Similar changes were also observed in labeled ICCs (Fig. 2E,F and G). In addition to studying pancreas-specific gene expression in these ICCs, their insulin-synthesizing capacity was also investigated by measuring cellular C-peptide to verify and validate their functionality. The cellular C-peptide content per ICC was 0.89 ± 0.17 pmol for non-labeled ICCs and 1.08 ± 0.25 pmol for labeled ICCs (Fig. 2H). This result is consistent with the insulin gene expression analysis data described above.

### *In vitro* MRI of SPION-labeled PPCs

**Figure 3:**
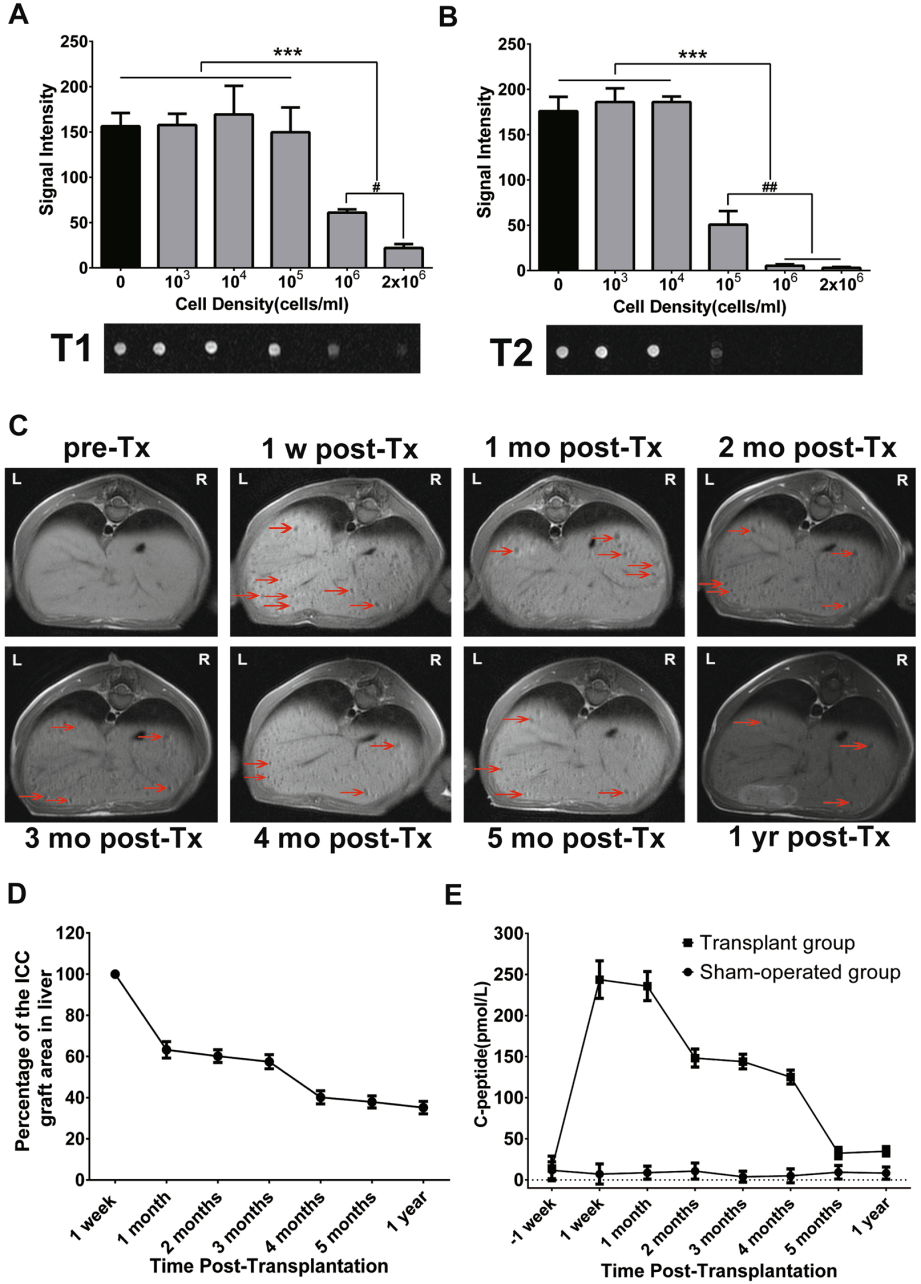
(**A** and **B**) Bar graphs showing the signal intensity values measured in gelatin phantoms containing SPION-labeled cells after overnight incubation with 100 μg Fe/ml SPIONs at 37 °C. The graphs (top) and corresponding MR images (bottom) were obtained by T1-weighted imaging (**A**) and T2-weighted imaging (**B**). Quantitative analysis revealed a significant decrease in signal intensity of more than 10^6^ cells/ml on T1-WI or more than 10^5^ cells/ml on T2-WI. The results are shown as the mean ± SD, n = 3 (independent experiments); ***P < 0.001, ^#^ *P* < 0.05, ^##^ *P* < 0.01. (**C**) Longitudinal MR imaging of labeled autologous ICCs transplanted via the intraportal route in diabetic monkeys. The arrows indicate hypointense spots, which are representative of labeled ICCs. (**D**) Semiquantitative assessment of graft survival time revealed a significant drop in graft areas from week 1 to month 1 and from month 3 to month 4 after transplantation. (**E**) Fasting C-peptide levels were measured in the sham-operated group or the transplant group throughout the follow-up period. The monkeys engrafted with autologous PPC-derived IPCs showed a significant increase in fasting C-peptide levels after transplantation, whereas the sham-operated animals did not.

To detect dynamic changes in transplanted ICCs by *in vivo* MRI, we first confirmed that the clinical 1.5T MR imaging unit was sufficiently sensitive to detect the change in signal intensity caused by SPION-labeled cells and establish the correlation between the labeled cell number and MRI signal intensity. We performed MR imaging of magnetically labeled cells at different cell densities. T1- and T2-weighted MRI revealed a cell dose-dependent decline in signal intensity *in vitro*. Moreover, T2-weighted MRI was more sensitive than T1-weighted MRI in detecting SPION-induced changes in signal intensities. Our analysis demonstrated that 1 × 10^5^/ml SPION-labeled cells resulted in a 70% loss of signal intensity on T2-weighted MRI compared with the control but did not induce any significant change in signal intensity on T1-weighted MRI. Furthermore, when the labeled cells reached densities of 1 × 10^6^ cells/ml or 2 × 10^6^ cells/ml, a greater than 97% loss of signal intensity on T2-weighted MRI was observed, whereas losses of signal intensity of only 60% and 86%, respectively, were noted on T1-weighted MRI (Fig. 3A and B).

### Long-term monitoring of dynamic changes in blood C-peptide levels and *in vivo* MRI of SPION-labeled ICCs

To determine whether noninvasive monitoring of SPION-labeled cells using MRI is a more sensitive method to determine the survival of grafted ICCs than the conventional measurement of blood C-peptide levels in diabetic monkeys, we performed a one-year follow-up monitoring study after transplantation. Although intraportal transplantation of ICCs did not reverse hyperglycemia in any of the six recipients, insulin synthesis and secretion were partially restored. Fasting C-peptide levels were almost undetectable in diabetic monkeys prior to transplantation, but at 1 week after intrahepatic autologous IPC infusion, a significant elevation of fasting C-peptide levels was detected, and average fasting C-peptide levels increased up to 243 pmol/ml, approximately 50% of normal fasting plasma C-peptide levels. Moreover, there was no significant decline in fasting C-peptide levels until 2 months post-transplantation. No significant differences in fasting C-peptide levels were observed in the sham-operated group before and after transplantation (Fig. 3E).

To assess the feasibility of monitoring the fate of transplanted ICCs *in vivo* in real time by MRI and the temporal relationships among MRI signal intensity changes, C-peptide levels, and the viability of grafted cells, we performed time-point MR imaging of transplanted ICCs up to one year after autologous transplantation. MRI revealed that the intrahepatically transplanted SPION-labeled ICCs caused obvious signal loss and appeared as hypointense spots of different sizes on different MR imaging sequences. These hypointense spots were dispersed in the liver lobe (Fig. 3C). However, the image quality differed significantly between the T1- and T2-weighted sequences. The contrast between the graft and surrounding liver tissues was greater in the T1-weighted sequence than the T2-weighted sequence, whereas the T2-weighted sequence was more sensitive than the T1-weighted sequence in revealing SPION-labeled ICCs *in vitro* (Figure S2). We further investigated whether T1-weighted MRI could be used to accurately predict post-transplantation outcome. As previously reported, the total area of hypointense spots was more closely correlated with the blood glucose levels of the recipients^23^. Here, we analyzed the temporal relationship between the changes in the total area of hypointense spots in the T1-weighted sequence and blood C-peptide levels in the recipients. The analyses demonstrated that the total area of hypointense spots decreased 37% from week 1 to month 1 and 30% from month 3 to month 4 after transplantation in the T1-weighted sequence. More importantly, in all six monkeys, the loss of the graft area observed on T1-weighted MRI preceded the decline in fasting C-peptide levels. The loss of graft area was evident from month 1 to month 2 and from month 4 to month 5 after transplantation (Fig. 3D and E). These results indicate that *in vivo* MR imaging is more useful for the timely detection of ICC graft loss in the liver than serum C-peptide monitoring.

### Histological and immunohistological analyses of intraportally transplanted ICCs

**Figure 4:**
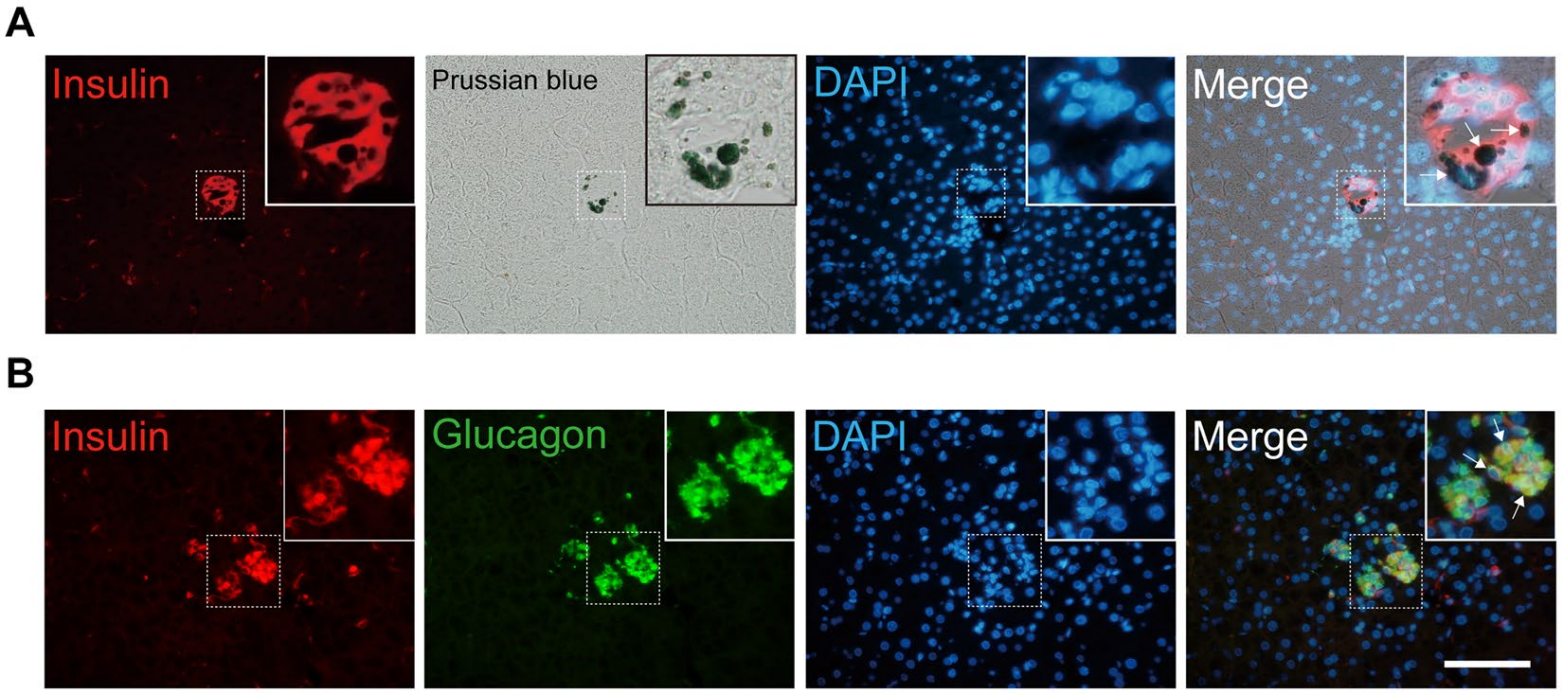
Histology and immunochemical staining of transplanted ICCs. (**A**) The colocalization of insulin activity and SPIONs was verified by double staining with immunofluorescence staining with anti-insulin antibodies (red fluorescence) and Prussian blue staining for iron (blue). White arrows showed where iron particles were located within the insulin-positive cells. (**B**) Immunostaining for insulin (red fluorescence) and glucagon (green fluorescence) revealed the expression of islet hormones in the transplanted ICCs. Note that some of insulin-positive cells co-expressed glucagon (indicated by white arrows). Cell nuclei were visualized using DAPI staining (blue fluorescence). Insets showed magnified views of indicated area. Scale bar represents 100 μm.

One year after the autotransplantation of the SPION-labeled ICCs, the colocalization of SPIONs and insulin activity was confirmed by double staining with Prussian blue staining and insulin immunostaining (Fig. 4A), which indicated that SPIO-labeled PPCs differentiated into IPCs. Further dual immunofluorescence staining demonstrated that transplanted ICCs expressed both insulin and glucagon (Fig. 4B). Interestingly, we found that some of the insulin-positive cells were costained for glucagon, suggesting that these cells were still immature endocrine cells.

To further investigate the distribution of iron oxide particles in the liver macrophages, double staining for iron and CD68 (a marker of macrophage) were performed on liver sections. The results demonstrated that few CD68 positive cells were iron-positive (Figure S3).

**Figure 5:**
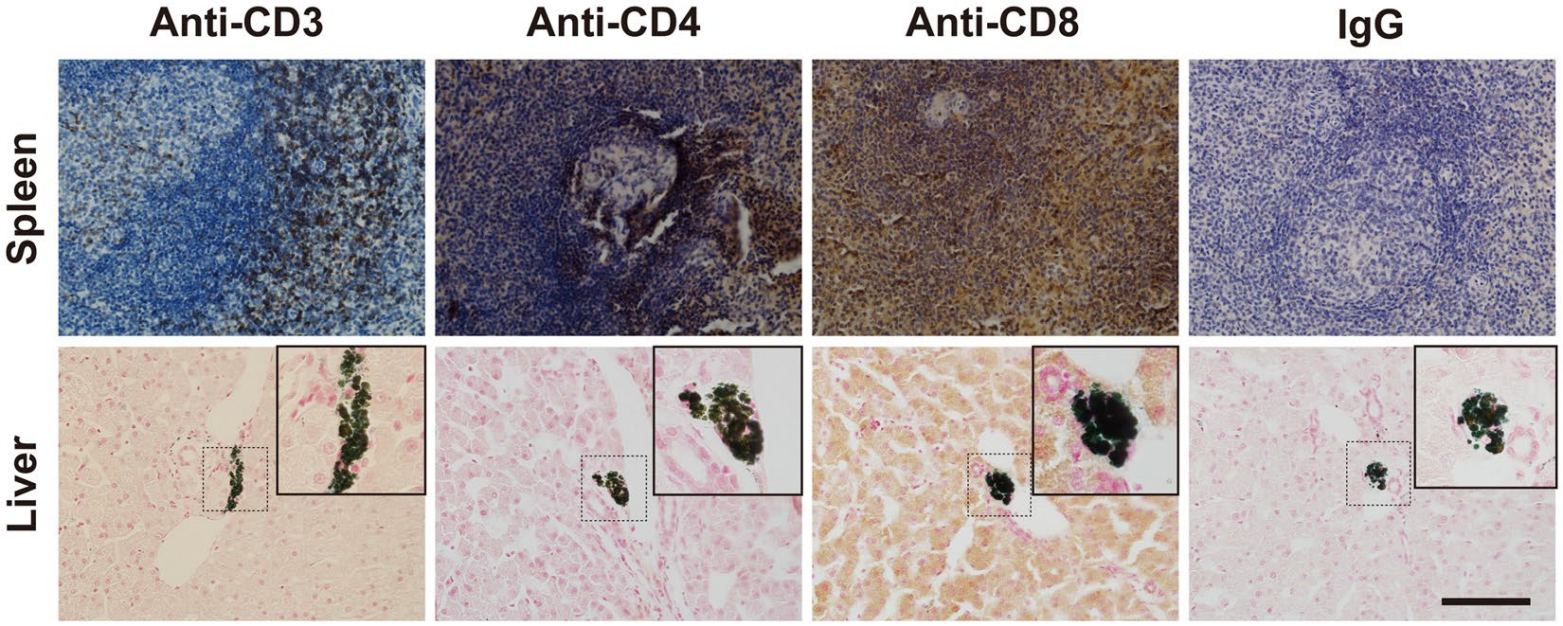
Immunogenicity of autografted ICCs in diabetic monkeys. Infiltration of T cells was not detected within and surrounding the transplanted ICCs on the monkey liver sections. T cells were identified by immunostaining with anti-CD3, anti-CD4 and anti-CD8 antibodies. The upper row represents the monkey spleen tissue sections that served as positive controls for CD3, CD4 and CD8. Cell nuclei were visualized using hematoxylin or nuclear fast red counterstain. Insets showed magnified views of indicated area. Scale bar represents 100 μm.

To examine the immunogenicity of PPC-derived ICCs in intraportal autologous transplantation, we examined T-cell infiltration via an immunostaining assay for CD3, CD4 and CD8 in recipient livers at the end of the observation time. The results showed no detectable T-cell infiltration in and surrounding the grafted ICCs (Fig. 5). These findings suggested that the PPC-derived ICCs did not cause an immune rejection response in autologous transplantation.

## Discussion

The major obstacles to islet transplantation are the shortage of donors, immune rejection and side effects from immunosuppression^24^. Induced pluripotent stem cells hold potential for autologous transplantation using insulin-producing cells derived from a diabetic patient’s own cells. However, previous studies have indicated that non-immunological graft loss occurs even in autologous islet transplantation and that islet graft function slowly deteriorates after transplantation^25, 26^. Therefore, it is important to longitudinally monitor islet graft loss in a timely manner using noninvasive methods. Although a decrease in blood insulin and C-peptide levels is a good indicator of islet dysfunction, the major drawback of these analyses is the temporal delays in response to pancreatic β-cell loss^21^. A clinically useful method should enable close monitoring of the status of transplanted islets to enable timely detection of graft loss and early intervention to help recipients maintain stable blood glucose levels.

Several groups have demonstrated the feasibility of imaging pancreatic islets by labeling with SPIONs in mouse, rat and monkey models of T1D^12, 18, 27^. However, few studies have considered the temporal relationship between the loss of SPION-labeled IPCs on MRI and the decrease in blood insulin or C-peptide levels, and few have demonstrated that SPION-based MR tracking is more sensitive for timely detection of IPC loss than measuring blood insulin or C-peptide levels. In the present study, we differentiated SPION-labeled monkey PPCs to IPCs using a previously described method^22, 28^, and we transplanted cultured autologous IPCs into the liver of STZ-induced diabetic monkeys without immunosuppression. To rule out the possibility that the observed increase in blood C-peptide levels was due to spontaneous β-cell regeneration, insulin immunostaining was performed in pancreas sections. In agreement with previously reported results^29, 30^, no spontaneous β-cell regeneration was observed in the pancreatic tissues following the injection of a single high dose of STZ, which eliminated nearly all pancreatic β-cells in the diabetic animals. Blood C-peptide levels were almost non-detectable even using ultra-sensitive ELISA assays. It would be helpful to observe the subtle changes in blood C-peptide levels caused by cell transplantation.

Our examination of the biological characteristics of PPCs revealed that PPCs shared common surface markers with bone marrow-derived MSCs but did not express endocrine markers, including PDX-1 and insulin. Interestingly, the PPCs expressed the transcription factor Isl-1, which is well-known for its role in the development of pancreatic endocrine cells^31^. Our data suggested that PPCs are a type of pancreas-derived mesenchymal stem cell. Although the postnatal origins of pancreatic β-cells *in vivo* remain controversial^32–36^, many studies have demonstrated that pancreatic stem cells can differentiate into IPCs upon exposure to appropriate stimuli *in vitro* ^37–40^. In this study, autologous PPCs were employed as a model to investigate whether SPION-based MR tracking can be used for the timely detection of the loss of grafted stem cell-derived IPCs.

Although SPIONs have been used to label mouse embryonic stem cell (ESC)-derived IPCs for MRI-based monitoring *in vivo*, no studies have examined whether SPION labeling has undesirable effects on the differentiation of pancreatic progenitor cells into IPCs. Our results demonstrated that the labeling process did not disturb the pancreatic differentiation potential of PPCs and did not affect insulin synthesis in the differentiated IPCs. These results suggest that SPIONs can be used to label PPCs for *in vivo* tracking purposes before the induction of the differentiation of PPCs into IPCs *in vitro*. Undoubtedly, this capability is important for longitudinal MR tracking of transplanted IPCs in the liver. In previous studies^23, 27^, the majority of cells in the core region of isolated islets were not labeled with SPIONs, probably due to the difficulty of dispersing SPIONs into the core of the islets. This low level of core labeling might result in a false sharp decrease in the total areas of hypointense spots on MR images even when only peripheral SPION-labeled islet cells are destroyed. Accordingly, at a later stage, when the non-labeled cells in the core area of islets are lost, no changes in MR signal intensities would be detected, potentially leading to misinterpretation of the relationship between islet detection by MRI and islet function at later time points after transplantation^41^. In a recent preclinical study using non-human primates (NHPs)^21^, Wang *et al*. demonstrated that the decline in graft volume in MR images presented as different decline patterns between two diabetic monkeys after receiving allogeneic islet transplantation. We speculate that the different SPION labeling efficiencies in the core cells of isolated islets might be responsible for this heterogeneity. In the current study, we observed that the decline in the total area of hypointense spots occurred before changes in blood C-peptide levels, which may be caused by a gradual release of C-peptide from the dying IPCs. These results are similar to previous findings^21^ and may be conducive to designing timely therapeutic interventions for the protection of implanted IPCs from further damage. We believe that our observation has important implications for clinical applications of autologous stem cell transplantation for T1D treatment for the following reasons. First, in all six experimental monkeys, a rapid decrease in the total area of the graft signals was observed on T1-weighted imaging in the first month after autotransplantation. Previous studies have indicated that embolization of the portal vessels by the grafted islets can cause local hypoxia, production of pro-inflammatory cytokines surrounding liver tissue, and, subsequently, injury and apoptosis of the grafted islet β-cells^42, 43^. The immediate blood-mediated inflammatory reaction (IBMIR) might also contribute to the early destruction of grafted islets during autologous islet transplantation^44^. In this study, we reasoned that ischemic liver injury and IBMIR may together lead to early graft loss. A timely strategy aimed at preventing local hypoxia and IBMIR reactions at an early stage of transplantation would be helpful to improve the survival and function of autologous stem cell-derived IPCs. Second, the similarity in the pattern and magnitude of the changes in the total areas of hypointense spots or in blood C-peptide levels was observed in all recipients after transplantation. This result indicated that these grafts might have suffered from similar damage; this pattern of graft destruction in autologous stem cell transplantation has not been observed in allogeneic islet transplantation studies. Our findings indicate that a standard therapeutic intervention strategy could be developed for preventing graft loss in autologous transplantation. Third, and more importantly, our study showed that T1-weighted MRI is sufficiently sensitive to detect changes in hypointense spots and thus may represent an accurate and easy-to-perform method to monitor islet graft loss in clinical practice.

Notably, MRI follow-up revealed a significant decrease in the total areas of hypointense spots from month 3 to month 4 after transplantation. Multiple mechanisms might contribute to this late graft loss, including hyperglycemia-induced apoptosis, delayed angiogenesis, and immunological and non-immunological insults^45–47^. Although it has been generally assumed that adult autologous stem cells do not cause an immune rejection response, it is not clear whether prolonged exposure to xenogenic proteins within the culture milieu during expansion and differentiation alters the immunogenicity of these cells^48, 49^. Immunohistochemical staining of the monkey liver sections in the present study showed no T-lymphocyte infiltration in and surrounding the graft, suggesting an absence of an immune response. These results indicate that the expansion and differentiation of adult stem cells do not affect the immunogenicity of these cells following transplantation to the liver. Therefore, the underlying mechanism leading to this late graft loss requires further investigation.

In conclusion, we have provided experimental evidence supporting a clinically feasible MRI-based method to monitor IPCs following transplantation. Our findings suggest that labeling of pancreatic progenitor cells with SPIONs using the methods described here is not toxic to cells and does not inhibit their ability to differentiate to IPCs. Furthermore, the results of *in vivo* MRI, blood serum tests and histological analyses suggest that the labeling and detection strategy described in this study can reasonably be used to monitor the function and viability of ICC grafts in a timely and reliable manner, thus providing a longitudinal noninvasive tool for clinicians to follow the fate of grafted IPCs using clinical MRI technology.

## Methods

### Ethics statement

The animal experiments were conducted at the Primate Research Center of Wincon TheraCells Biotechnologies Co. Ltd (Guangxi, China), which is an AAALAC-accredited non-human primate research facility. The experimental protocol was approved by the Institutional Animal Care and Use Committee of Wincon TheraCells Biotechnologies. All experimental procedures were conducted in accordance with the *National Institute of Health Guide for the Care and Use of Laboratory Animals* (NIH Publications No. 80–23, revised 1996).

### Animals

Pathogen-free male adolescent cynomolgus monkeys (2–3 years old, 2.5–4 kg) were used in this study. All animals had continuous access to water and were fed a “primate diet” twice daily, supplemented with fresh fruit and vegetables (more details are available upon request). The animals were conditioned for a minimum of 1 month prior to the start of the experiment. The experimental paradigm is illustrated in Fig. 1A. The schematic diagram depicts the timeline of experiments starting from the induction of diabetes to the euthanasia of the animals for histological analysis. Briefly, a small portion of the pancreas was removed surgically 3 months after STZ injection, and PPCs were isolated from the pancreata, expanded for 8 passages in growth medium over 2.5 months, and labeled with SPIONs. The labeled PPCs were then differentiated into ICCs in induction medium. Autologous ICCs labeled with SPIONs were subsequently transplanted intrahepatically, and the survival and function of the grafted ICCs were monitored and followed for up to one year. At the end of the observation time, histological analyses of the liver and pancreas were performed after euthanasia.

### Type 1 diabetic monkey model

All monkeys were fasted for 12–15 h before STZ administration, and the animals were anesthetized with ketamine (10 mg/kg body weight) and atropine (0.04 mg/kg). A single dose of streptozotocin (100 mg/kg body weight) was administered immediately after dissolution via the saphenous vein over a period of 5 minutes. To avoid metabolic dysfunction, the diabetic monkeys were treated with two injections of insulin guided by capillary glucose levels daily. The diabetic monkeys were then randomly allocated into two groups, the PBS-injected sham group (sham-operated group, n = 3) and IPC autotransplant group (transplant group, n = 6).

### Isolation of diabetic monkey islets and cell culture

PPCs were isolated as reported previously^22^ and propagated in RPMI 1640 medium (Life Technologies, Carlsbad, CA, USA) supplemented with 10% FBS (Life Technologies), 20 ng/ml bFGF, and 20 ng/ml EGF (both from R&D Systems, Minneapolis, MN, USA).

### Flow cytometry analysis

For flow cytometry analysis of PPCs, the cells were harvested, washed with PBS, resuspended in PBS, and stained with fluorescent antibodies for 60 min at 4 °C in the dark. We used CD73-PE (eBioscience, San Diego, CA, USA), CD90-PE (eBioscience), CD105-PE (eBioscience), CD34-FITC (eBioscience), CD45-PE (eBioscience), and HLA-DR-FITC (eBioscience) to characterize the expanding PPCs. Isotype-matched antibodies served as controls.

### *In vitro* cell labeling

To label the PPCs, we combined SPIONs (Feridex) at different iron concentrations with 1.5 μg/ml poly-L-lysine (PLL) in the growth medium and then gently mixed the medium on a shaker for 1 hour. The medium containing the SPION-PLL complex was added to the cultured PPCs. After overnight culture, the labeled PPCs were washed 3 times with phosphate buffer solution (PBS) and used in either *in vitro* or *in vivo* experiments.

### Cell viability assays

Cell viability was assessed using the MTS assay and MTS reagent (CellTiter 96® AQueous One Solution Cell Proliferation assay, Promega, Madison, WI, USA). Briefly, 4 × 10^3^ PPCs growing exponentially were seeded in 96-well culture plates. The following day, different concentrations of SPIONs were added to each well, and the plate was re-incubated at 37 °C in 5% CO_2_. After a 48-h incubation at 37 °C, 20 µl of MTS was added to each well, and the samples were incubated for a further 3 h at 37 °C. The plates were analyzed on a Bio-Rad Model 680 Micro plate Reader at 490 nm.

### MRI of labeled PPCs

Uniform gel suspensions (8% gelatin) were prepared with cell concentrations of 0, 1.0 × 10^3^, 1.0 × 10^4^, 1.0 × 10^5^, 1.0 × 10^6^ and 2.0 × 10^6^ cells/ml, and the signal intensity on T1- and T2-weighted images was measured using a 1.5-T clinical MRI system.

### ICC differentiation studies

To initiate cell differentiation, labeled and non-labeled PPCs were first transferred to Petri dishes containing DMEM/F12 (5.6 mM glucose) supplemented with 2% B27 (Invitrogen), bFGF (20 ng/ml, R&D Systems) and EGF (20 ng/ml, R&D Systems). Islet-like cell clusters (ICCs) spontaneously formed in 48–72 h. The ICCs were further induced into IPCs in serum-free media containing B27, 0.05% BSA, hepatocyte growth factor (HGF) (10 ng/ml, R&D Systems), betacellulin (500 pM, R&D Systems), Exendin-4 (10 nM, Sigma-Aldrich, St Louis, MO, USA) and nicotinamide (10 mM, Sigma-Aldrich). C-peptide was extracted from PPC-derived ICCs using acid/ethanol at 4 °C overnight, and the concentration was measured using a human C-peptide ELISA kit (Mercodia, Uppsala, Sweden).

### Real-time quantitative PCR

Total RNA was isolated from cultured PPCs and from labeled and non-labeled ICCs using TRIzol (Life Technologies). First-strand cDNA was synthesized from RNA samples using the SuperScript® III Reverse Transcriptase Kit (Life Technologies). Real-time PCR analysis of Isl-1, Pdx-1 and insulin expression was performed using an ABI 7300 Real Time PCR System (Applied Biosystems, Foster City, CA, USA). Data were normalized to the housekeeping gene β-actin. The primer sequences are listed in Supplementary Table S1.

### Autologous ICC transplantation in diabetic monkeys

All animals were fasted for 12 h before surgery. The animals were initially sedated with a combination of ketamine HCl (10 mg/kg i.m.) and atropine sulfate (0.04 mg/kg i.m.) and were then maintained in a general anesthetic state with isoflurane (1–1.5% in 100% O_2_). During surgery, the body temperature of the animals was maintained using a heating pad. Animals were routinely administered intra-operative fluid and continuously monitored for cardiac electrical activity, blood pressure, respiratory rate, oxygen saturation, and end tidal CO_2_. An 18-gauge Teflon catheter was inserted into the jejunal mesenteric vein via laparotomy and advanced into the portal vein to permit the infusion of approximately 60,000 ICCs into the liver. After ICC infusion, the abdomen was closed, and the monkeys were monitored closely until fully awake.

### MRI of transplanted labeled ICCs *in vivo* in diabetic monkeys

The diabetic monkeys underwent MR imaging of the liver before transplantation and at 1 week, 1 month, 2 months, 3 months, 4 months, 5 months and 1 year after SPION-labeled ICC transplantation. All MR imaging experiments were performed using a 1.5-T clinical MRI system. The following imaging sequences were applied: (*a*) coronal FSE T1-weighted images obtained using the following parameters: TR/TE = 600/15 ms, slice thickness = 5.0 mm, FoV = 160 × 160 mm^2^, spacing = 1.0 mm, echo train length = 4.0, and bandwidth = 31.25 kHz; and (*b*) coronal T2-weighted images obtained using the following parameters: TR/TE = 3000/102 ms, slice thickness = 5.0 mm, FoV = 160 × 160 mm^2^, spacing = 1.0 mm, echo train length = 17.0, bandwidth = 31.25 kHz, and NEX = 4.0. Analyses were performed by two authors (X.S., G.S.) using ImageJ software and Image-Pro Plus software. The areas of hypointense spots were measured for all slices acquired throughout the liver.

### Histology and immunostaining analyses

Fresh tissue and cultured cells were fixed in 4% paraformaldehyde, and immunostaining was performed using primary antibodies prepared in different species: rabbit anti-nestin (1:200, Chemicon International, Temecula, CA, USA), rabbit anti-Isl-1 (1:200, Chemicon International), mouse anti-Vimentin (1:500, Chemicon International), rabbit anti-PDX-1(1:500, Chemicon International), mouse anti-glucagon (1:1000, Sigma-Aldrich), guinea pig anti-insulin (1:100, Zymed Laboratories, South San Francisco, CA, USA), mouse anti-C-peptide (1:250, Chemicon International), rabbit anti-CD3 (ready to use, ZSGB-BIO, Beijing, China), mouse anti-CD4 (1:60, Dako, Glostrup, Denmark), mouse anti-CD8 (1:80, DAKO) and mouse anti-CD68 (1:80, DAKO). Binding of the primary antibody was visualized by either the immunofluorescence technique or an HRP enzymatic reaction using 3,3′-diaminobenzidine (DAB) as the chromogen. For Prussian blue staining, fixed cells and tissue sections were incubated in a 1:1 mixture of 4% potassium ferrocyanide and 4% HCl for 50 minutes. DAPI or nuclear fast red were used as nuclear counterstains. Representative images were captured using an Olympus BX53 Microscope or NIKON2000U microscope.

### Electron Microscopy

Labeled and non-labeled PPCs were fixed in 3% neutral phosphate-buffered glutaraldehyde and postfixed with 1% osmium tetroxide in 0.1 M phosphate buffer (pH 7.4) for 1 h. Then, the samples were dehydrated in a graded ethanol series and embedded in epoxy resin (Epon 812). Ultrathin sections (70 nm thick) were cut and stained with uranyl acetate and lead citrate. The sections were examined by transmission electron microscopy (JEM-1230 operated at 80 kW) and photographed.

### Statistical analysis

The results were expressed as the mean ± standard deviation (SD). Data analyses were performed using SPSS version 19.0 (SPSS Inc., Chicago, IL) and GraphPad Prism version 6.00 for Windows (La Jolla, CA, USA). Continuous variables were compared using ANOVA. *P* values of <0.05 were considered statistically significant.

## Electronic supplementary material

Supplementary Information
