# Supplementary material for: MRI tracking of autologous pancreatic progenitor-derived insulin-producing cells in monkeys
Source: Sci Rep. 2017 May 31;7:2505. doi: 10.1038/s41598-017-02775-0 (PMC5451407; doi:10.1038/s41598-017-02775-0)
Supplement: Supplementary file 1 — Supplementary Information [file 41598_2017_2775_MOESM1_ESM.doc]

**MRI tracking of autologous pancreatic progenitor-derived insulin-producing cells in monkeys**

Chunlin Zou^1,2,3^*, Yi Lu^1^, Xiahong Teng^1^, Shuyan Wang^2,3^, Xiaoting Sun^1^, Fen Huang^1,4^, Guannan Shu ^1^, Xin Huang^1^, Hongwei Guo^1^, Zhiguo Chen^2,3^, Jian Zhang^1^, Yu Alex Zhang^2,3^*

^1^ Center for Translational Medicine, Guangxi Medical University, Guangxi, P.R. China

^2^ Cell Therapy Center, Xuanwu Hospital, Capital Medical University, Beijing, P.R. China

^3^ Key Laboratory of Longevity and Ageing-related Diseases, Ministry of Education, Guangxi Medical University, Guangxi, P.R. China

^4^ Guangxi Nanning Wincon TheraCells Biotechnologies Co., Guangxi, P.R. China

*Correspondence:

Yu Alex Zhang, Ph.D., Cell Therapy Center, Xuanwu Hospital, Capital Medical University, Beijing, China. Telephone: 8610-63184557, Fax: 8610-83198889, Email: [yaz@bjsap.org](mailto:yaz@bjsap.org)

Chunlin Zou, Ph.D., Center for Translational Medicine, Guangxi Medical University, Guangxi, P.R. China. Telephone: 86771-5300270, Fax: 86771-5310865, Email: zouchunlin@sohu.com

**Supplementary Figures**

Figure S1. Electron photomicrographs of non-labeled (A) and labeled PPCs (B and C). (B) The arrowheads indicate endosomal vesicles containing SPIONs inside the cytoplasm of labeled cells. The SPIONs displayed high electron density and a unique granular morphology. N, nucleus; M, mitochondria; Ly, lysosome. (C) The arrow indicates that the PPC was phagocytizing the Feridex-PLL complexes. Scale bars represent 2 μm in A and B, 5 μm in C.

Figure S2. The T1-weighted MRI scan provided better contrast for the grafted ICCs than the T2-weighted MRI scan.

Figure S3. A representative double staining for iron (blue) and CD68 (green fluorescence) on the monkey liver section. Few CD68+Prussian blue double positive cells were observed (indicated by red arrow). Cell nuclei were visualized using DAPI staining (blue fluorescence). Scale bar represents 100 μm.

**Supplementary Figure S1**


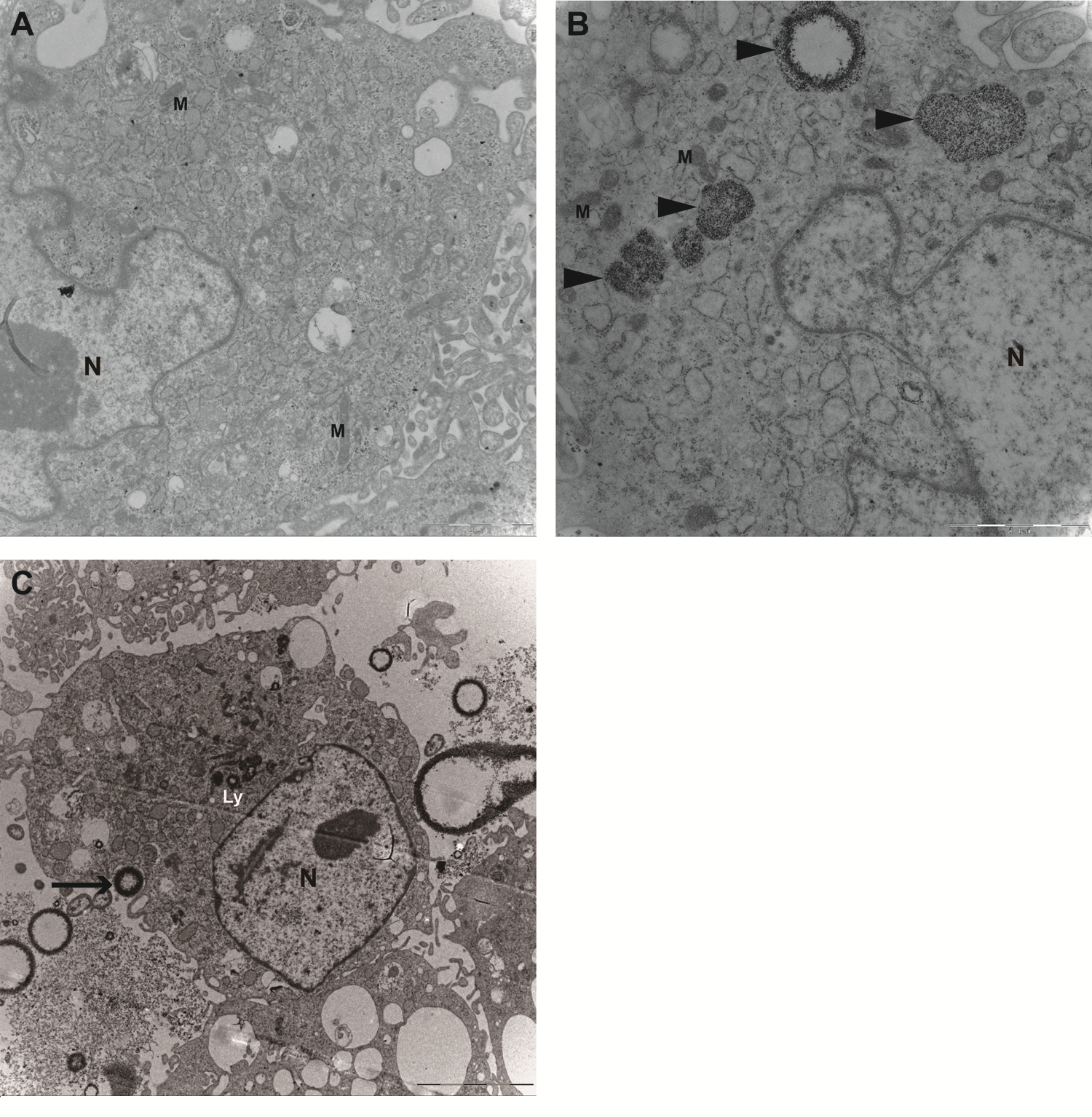


**Supplementary Figure S2**

**
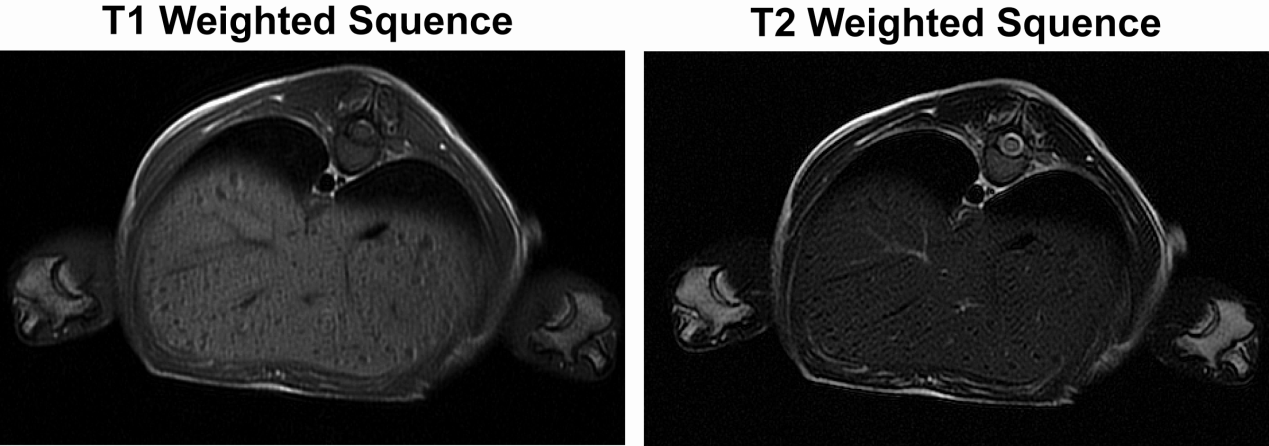
**

**Supplementary Figure S3**


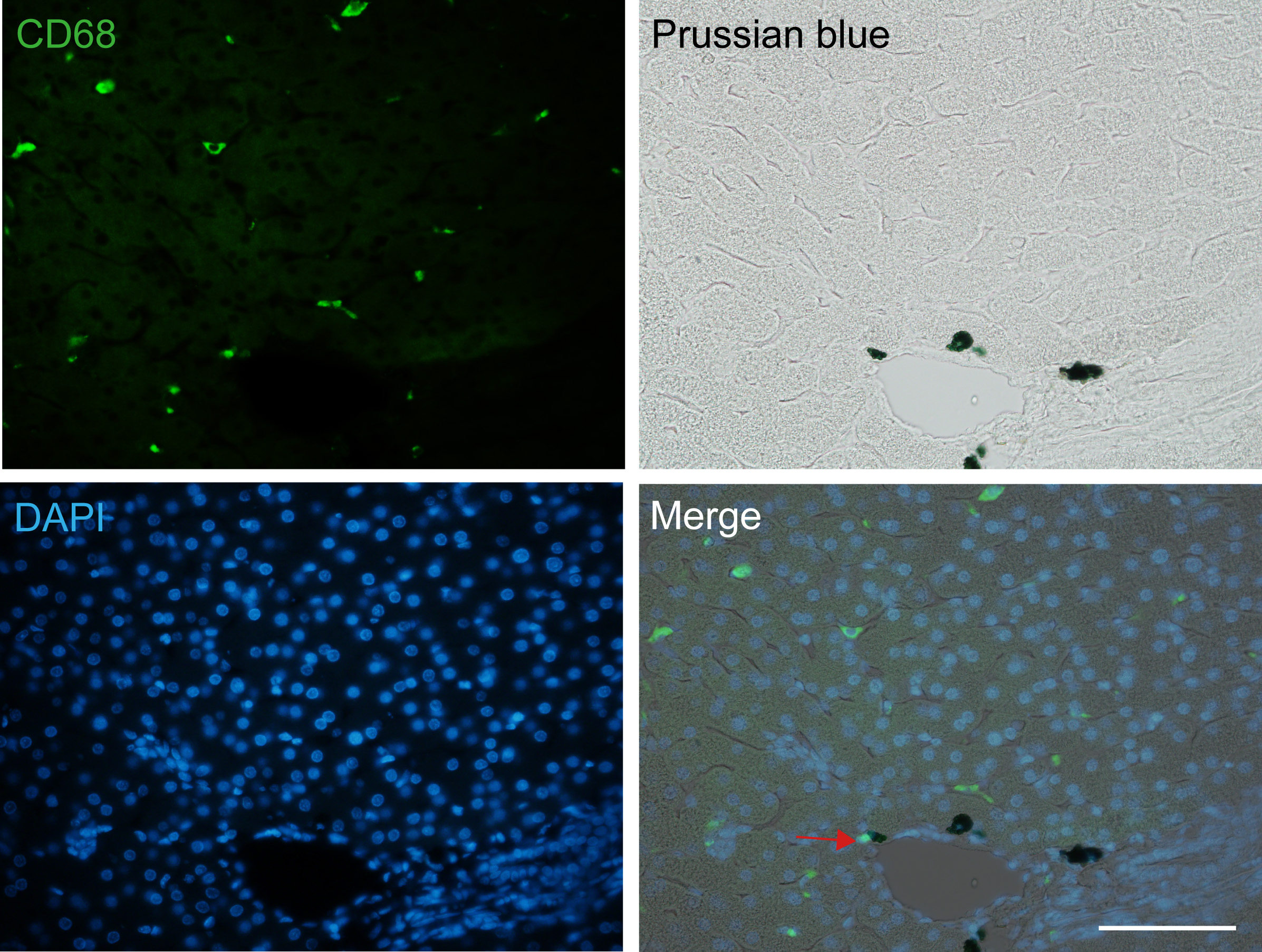


**Supplementary Table**

**Table S1 Sequences of specific RT-PCR primers**

| Gene Sense primer (5'→3') Antisense primer(5'→3') Product size(bp) |
| --- |
| Isl-1 TGTTTGAAATGTGCGGAGTG GTTCTTGCTGAAGCCGATG 144  PDX-1 GGAGCCGGAGGAGAACAAG CTCGGTCAAGTTCAACATGACAG 139  Insulin GCAGCCTTTGTGAACCAACAC CCCCGCACACTAGGTAGAGA 67  β-actin TGTCCACCTTCCAGCAGATGT CGGACTCGTCATACTCCTGCTT 51 |
